# The association of the treatment with glucagon-like peptide-1 receptor agonist exenatide or insulin with cardiovascular outcomes in patients with type 2 diabetes: a retrospective observational study

**DOI:** 10.1186/s12933-015-0178-3

**Published:** 2015-01-24

**Authors:** Sanjoy K Paul, Kerenaftali Klein, David Maggs, Jennie H Best

**Affiliations:** Clinical Trials & Biostatistics Unit, QIMR Berghofer Medical Research Institute, 300 Herston Road, Herston, QLD 4006 Brisbane, Australia; Statistics Unit, QIMR Berghofer Medical Research Institute, Brisbane, Australia; Bristol-Myers Squibb, San Diego, CA USA; University of Washington, Seattle, WA USA

**Keywords:** Exenatide, Insulin, Macrovascular outcomes, Type 2 diabetes, Pharmaco-epidemiology

## Abstract

**Background:**

To evaluate the association of treatment with glucagon-like peptide-1 (GLP-1) receptor agonist exenatide and/or insulin on macrovascular outcomes in patients with type 2 diabetes (T2DM).

**Methods:**

We conducted a retrospective longitudinal pharmaco-epidemiological study using large ambulatory care data to evaluate the risks of heart failure (HF), myocardial infarction (MI) and stroke in established T2DM patients who received a first prescription of exenatide twice daily (EBID) or insulin between June 2005 and May 2009, with follow-up data available until December 2012. Three treatment groups were: EBID with oral antidiabetes drugs (OADs) (EBID, n = 2804), insulin with OADs (Insulin, n = 28551), and those who changed medications between EBID and insulin or had combination of EBID and insulin during follow-up, along with OADs (EBID + insulin, n = 7870).

Multivariate Cox-regression models were used to evaluate the association of treatment groups with the risks of macrovascular events.

**Results:**

During a median 3.5 years of follow-up, cardiovascular event rates per 1000 person-years were significantly lower for the EBID and EBID + insulin groups compared to the insulin group (HF: 4.4 and 6.1 vs. 17.9; MI: 1.1 and 1.2 vs. 2.5; stroke: 2.4 and 1.8 vs. 6.1). Patients in the EBID/EBID + insulin group had significantly reduced risk of HF, MI and stroke by 61/56%, 50/38% and 52/63% respectively, compared to patients in the insulin group (p < 0.01).

**Conclusions:**

Treatment with exenatide, with or without concomitant insulin was associated with reduced macrovascular risks compared to insulin; although inherent potential bias in epidemiological studies should be considered.

## Background

Treatment of type 2 diabetes mellitus (T2DM) needs to address disease progression and balance the pharmacological efforts that limit hyperglycaemia against the increased risks of adverse effects including hypoglycaemia and weight gain. As patients with T2DM experience increased risk of cardiovascular (CV) morbidity and mortality over time, treatment strategies should ideally also address CV risk factors including body weight, blood pressure and lipids [1,2]. Initially, most patients take oral antidiabetes drugs (OADs) to combat hyperglycaemia. Once OADs fail to control hyperglycaemia, transition to injection of exogenous long-acting insulin has been the standard next step, introducing challenges including labour-intensive management, hypoglycaemia and weight gain.

Since their introduction, incretin-based therapies including the oral dipeptidyl peptidase 4 (DPP-4) inhibitors and injectable glucagon-like peptide-1 receptor (GLP-1R) agonists have attracted attention as treatment alternatives with unique mechanisms of action [2-8]. GLP-1R agonists offer particular promise for patients with T2DM because of potential cardioprotective effects. A large body of pre-clinical and clinical data suggests reduced cardiovascular risk with GLP-1R agonists via mechanisms including reduced body weight and adiposity [9-14], decreased blood pressure (BP) [11,15], improved endothelial and myocardial function [16-18], improved functional recovery of failing and ischemic hearts, increased diuresis and natriuresis, and reduced circulating lipids [18-21]. In humans with T2DM and congestive heart failure, GLP-1R agonists were reported to improve myocardial function. In patients with low left ventricular ejection fraction (LVEF) after acute myocardial infarction, GLP-1 infusion improved LVEF and global and local wall indices [4,15]. Using a large patient-level data set from the United States, Best et al. reported significantly reduced rates of HF, MI and stroke in patients treated with exenatide for at least 31 days compared to those treated with insulin [22,23]. A recent epidemiological study based on Danish registry data reported significant risk reduction for mortality in patients receiving DPP-4 plus metformin (MET) compared to those treated with MET plus sulphonylurea (SU), although the observed risk reduction in patients treated with MET plus GLP-1R agonist was not significant [24]. However, the SAVOR-TIMI and EXAMINE study did not show any significant beneficial effects of treatment with DPP-4 inhibitors on macrovascular outcomes [25,26].

As a relatively new treatment for hyperglycaemia in patients with T2DM, the long-term effect of GLP-1R agonism on CV outcomes in clinical trials or actual practice has not been established. Whilst numerous large CV outcome trials are ongoing to evaluate CV outcomes with GLP-1R agonists in patients with T2DM, the results from such trials will not be known before 2015, and will not demonstrate the real-world effects of GLP-1R agonism.

The aim of our study was to evaluate CV risks in patients with T2DM treated with the GLP 1-R agonist exenatide twice daily (EBID) and/or insulin in a large, nationally-representative longitudinal ambulatory-care medical record database. Specific aims were to evaluate: (1) event rates of HF, MI, stroke in patients treated with EBID and conventional oral anti-diabetic drugs (OADs) (EBID group), insulin plus OADs (insulin group), and those who changed medications between EBID and insulin or had combination of EBID and insulin during follow-up, along with OADs (EBID + insulin); (2) risks of HF, MI, stroke and combined event of MI or stroke in patients belonging to EBID and EBID + insulin groups compared to those in the insulin group, adjusting for various risk factors.

## Methods

### Data source

Data for this study were obtained from General Electric Centricity database, which collate patient-level information from a network of outpatient offices in the United States in the Medical Quality Improvement Consortium (MQIC). MQIC practices use the Centricity Electronic Medical Record. These data represent more than 40 US states and a variety of ambulatory medical practices, including solo practitioners, community clinics, academic medical centres, and large integrated delivery networks. The database has been extensively used for academic research in the United States [27-29]. MQIC consists of over 17,000 physicians and other providers, of whom approximately 70% are primary care providers.

From more than 1.2 million patients with confirmed T2DM, a cohort of patients was selected who received first prescription of EBID or insulin between June 2005 and May 2009, with follow-up data available till December 2012. The cohort selection criteria included: (1) age ≥ 18 years at the start date of the cohort (index date), (2) no missing data on age, sex, ethnicity, smoking status, and HbA1c at index date, (3) at least two consecutive prescriptions of EBID or insulin on and immediately after the index date, and (4) complete information on event dates for HF, MI and stroke following the index date along with International Classification of Diseases, Ninth edition (three-digit ICD-9 codes) for these events. Any event with an ‘undefined’ reference was excluded. Patients with any prescription for a DPP-4 inhibitor were excluded to avoid residual confounding effects of another incretin therapy.

Under the above selection criteria, we obtained a cohort of 183,125 patients who received the first prescription of insulin or EBID between June 2005 and May 2009. Owing to significant differences in the distributions of age and glycated haemoglobin (HbA1c) on index date between treatment groups, a new cohort of 39,225 patients was identified with matching for age and HbA1c at index date, belonging to three treatment groups: (a) insulin group, n = 28,551, (b) EBID group, n = 2,804, and (3) EBID + insulin group, n = 7,870. Patients in the EBID + insulin group could start either of the drugs on the index date and needed at least two prescriptions of the other drug during the follow-up. A Coarsened Exact Matching method (generalisation of the propensity score method) was used to create matched data in the treatment groups [30].

Baseline demographic and clinical information included age, sex, ethnicity, smoking status, duration of diabetes (DD), body mass index (BMI), and systolic and diastolic blood pressure. Laboratory measurements included measures of HbA1c within a 3-month window prior to the index date. Complete information on OADs, anti-hypertensive and cardioprotective medications (CPMs) over time were obtained along with dates of prescriptions. The CPMs included statins, angiotensin-converting enzyme inhibitors (ACE), angiotensin receptor blockers (ARB) and beta blockers. History of cardiovascular diseases and renal diseases (including ICD 9 codes 585.1 to 585.9) prior to index date and during follow-up were obtained.

This study was exempt from ethics approval from an institutional review board and informed consent because, according to the US Department of Health and Human Services Exemption 4 (CFR 46.101(b)(4)), the research involved the study of existing data, and the subjects could not be identified directly or through identifiers linked to the subjects.

### Statistical methods

The basic statistics on patients’ characteristics were presented by number (%), mean (standard deviation: SD) or median (inter-quartile range: IQR). Event rates per 1,000 person years were calculated for specific CV events along with their 95% confidence intervals (CI). The composite event of MI or stroke was defined as the first occurrence of either of MI or stroke during follow-up. The time to composite events was the time to the occurrence of first of the composite events. To evaluate the risks of CV events in patients in EBID and EBID + insulin groups compared to those in the insulin group, adjusted Cox regression models were fitted. Multivariable models included gender, ethnicity, age at the start of cohort, BMI, HbA1c, systolic and diastolic blood pressure on the index date, history of CV disease, any renal disease prior to index date or during follow-up, use of MET, SU, CPMs or antihypertensive medications, and the duration of diabetes (calculated at index date from the available disease onset days). Separate models were fitted for all patients, for patients without history of CV diseases and for patients without history of CV diseases and renal diseases before or after the index date. The proportionality assumption in the Cox regression models was tested, and stratified multivariable Cox regression models were fitted with age-quartiles as a stratifying factor. As the inclusion criteria in the cohort included availability of 2 prescriptions within 60 days of index date, the “time to event” for the survival analyses were adjusted accordingly to avoid possible “immortality bias”. As the treatment of either drug could start anytime in the EBID + insulin group, there is a potential for time bias in the context of survival analysis. This aspect was dealt with using a time-varying covariate. As a part of sensitivity analyses, separate risk evaluations were performed for patients taking metformin only (n = 26052), and also by baseline BMI categories.

The number of recorded prescriptions for EBID and insulin during follow-up were obtained, and the minimum duration of treatment with EBID and insulin were calculated assuming a prescription is for at least one month. As a part of sensitivity analyses, to evaluate the possible association of duration of treatments with the risk of events, separate multivariate regression models were fitted by adding the treatment duration variables to the aforementioned multivariate models. Separate subgroup analyses were also conducted among patients taking MET or SU and HbA1c above 58.5 mmol/mol (7.5%) and 63.9 mmol/mol (8%) at index date.

## Results

In the cohort of 39,225 patients, mean (SD) age was 58 (13) years, 46% were male, 44% were Caucasian, 51% were current or ex-smokers, and 12% had a CV disease history before initiating EBID or insulin. Overall, 4% (n = 1576) of patients had evidence of renal disease before initiation of EBID or insulin. The proportions of patients with renal disease during follow-up in insulin, EBID and EBID + insulin groups were 14.2%, 4.7% and 6.5%, respectively. The proportions of patients with BMI ≥30 kg/m^2^ (obese) were significantly higher (89%) in the EBID and EBID + insulin groups compared to the insulin group (65%, Table 1). The median HbA1c and mean systolic blood pressure (SBP) levels were similar between the groups at the start of the cohort. Median duration of diabetes at index date was 1 year in all groups, with proportions of patients with DD above 5 years in insulin, EBID and EBID + insulin groups at 6%, 8% and 11% respectively. Overall median follow-up time was 3.5 years.

**Table 1:** **Characteristics of patients at the initiation of EBID or insulin treatment**

	**Insulin**	**EBID**	**EBID + Insulin**
**N**	28551	2804	7870
**Male** ^**π**^	13562 (48)	1130 (40)	3401 (43)
**Age** ^**α**^	59 (14)	56 (11)	56 (11)
**Diabetes duration (year)** ^**β**^	1.0 (0, 1)	1.0 (0.2, 1.8)	1.0 (0.1, 2.0)
**Diabetes duration (year)** ^**α**^	1.2 (2.2)	1.6 (2.2)	1.8 (2.7)
**White Caucasian** ^**π**^	12145 (43)	1214 (43)	3898 (50)
**Black**	3482 (12)	119 (4)	445 (60)
**Hispanic**	956 (3)	36 (1)	103 (2)
**Ex-smoker** ^**π**^	8033 (33)	813 (35)	2452 (37)
**Current smoker** ^**π**^	4499 (19)	321 (14)	874 (13)
**BMI (kg/m** ^**2**^ **)** ^**α**^	34.1 (8.3)	38.8 (7.9)	38.6 (7.8)
**Overweight (BMI > 30 kg/m** ^**2**^ **)** ^**π**^	5759 (24)	245 (10)	691 (10)
**Obese** ^**π**^	15949 (65)	2186 (89)	6210 (89)
**SBP (mmHg)** ^**α**^	131 (19)	130 (16)	130 (16)
**DBP (mmHg)** ^**α**^	75 (11)	78 (10)	77 (10)
**HbA1c (mmol/mol)** ^**Ω**^	62.8	59.6	63.9
**HbA1c (mmol/mol)** ^**β**^	58.5 (50.8, 70.5)	56.3 (48.6, 66.1)	59.6 (50.8, 71.6)
**HbA1c ≥ 7 %** ^**π**^	19804 (69)	1708 (61)	5606 (71)
**LDL-C (mmol/L)** ^**α**^	5.34 (2.11)	5.40 (1.98)	5.16 (1.96)
**Triglyceride (mmol/L)** ^**β**^	8.16 (5.43, 12.32)	9.15 (6.43, 12.93)	9.27 (6.44, 13.43)
**Follow-up (year)** ^**β**^	3.4 (1.8, 4.5)	3.3 (1.3, 4.5)	3.5 (3.0, 4.8)
**Medication**			
**Metformin** ^**π**^	16549 (58)	2534 (90)	6969 (89)
**Sulfonylurea** ^**π**^	12291 (43)	1682 (60)	5309 (67)
**Thiazolidinedione** ^**π**^	8589 (30)	1348 (48)	4218 (54)
**ACE/ARB** ^**π**^	22210 (78)	1990 (71)	6116 (78)
**Beta Blocker** ^**π**^	15156 (53)	1108 (40)	3444 (44)
**Statin** ^**π**^	23037 (81)	2355 (84)	6964 (88)
**Anti-hypertensive** ^**π**^	24513 (86)	2339 (83)	6916 (88)
**Aspirin**	18683 (65)	1672 (60)	5467 (70)
**Disease history prior to index date**			
**Renal** ^**π**^	1187 (4.16)	57 (2.03)	332 (4.22)
**HF** ^**π**^	901 (3.16)	48 (1.71)	191 (2.43)
**MI** ^**π**^	286 (1.00)	22 (0.78)	73 (0.93)
**Stroke** ^**π**^	351 (1.23)	14 (0.50)	82 (1.04)
**IHD** ^**π**^	1865 (6.53)	184 (6.56)	702 (8.92)
**PVD** ^**π**^	581 (2.03)	37 (1.32)	170 (2.16)
**Angina** ^**π**^	160 (0.56)	24 (0.86)	70 (0.89)

^**α**^Mean (SD); ^**β**^ Median (Q1, Q3); ^**π**^N(%),^**Ω**^Mean.

Proportions of patients with previous and current (post-index) CV events along with rates (per 1000 person-years) of individual events are presented in Table 2. Rates of HF, MI, and stroke were higher among patients in insulin group compared to those in the EBID or EBID + insulin groups. The proportion of patients with history of HF was higher in the insulin group (3.2%) than in the EBID group (1.7%) and EBID + insulin group (2.4%). Among those without HF history (n = 38,085), rates of HF (per 1000 person-years) in insulin, EBID and EBID + insulin groups were 17.1, 4.1 and 5.7, respectively. Compared to insulin, patients in EBID and EBID + insulin groups had 66% (hazard ratio (HR) CI: 0.22, 0.52) and 60% (HR CI: 0.32, 0.50) less risk of HF, respectively, after adjusting for covariates (Table 3). Patients with a higher BMI (HR: 1.03, p < 0.01) and the history or incidence of renal disease during follow-up (n = 5952, HR: 2.11, p <0.01) had increased HF risks. Male patients had no increased risk of HF (HR: 1.10, p = 0.14). Current and ex-smokers had 50% and 38% increased risk of HF (p < 0.01 in both cases). Among patients without CV disease history (n = 34,672) and without history of renal disease (n = 33,744), similarly reduced risks of HF were observed in EBID and EBID + insulin groups.

**Table 2:** **Proportions of patients with cardiovascular events before initiation of treatment and during follow-up and the rates (per 1000 person years) along with 95% confidence intervals of cardiovascular events, by treatment groups**

		**Insulin**		**EBID**		**EBID + Insulin**	
		**Previous**	**Current**	**Previous**	**Current**	**Previous**	**Current**
**HF**	N (%)	901 (3.16)	2094 (7.33)	48 (1.71)	49 (1.75)	191 (2.43)	195 (2.48)
	Rate (CI)	-	17.87 (17.00, 18.78)	-	4.38 (3.17, 6.05)	-	6.13 (5.30, 7.07)
**MI**	N (%)	286 (1.00)	312 (1.09)	22 (0.78)	14 (0.50)	73 (0.93)	39 (0.50)
	Rate (CI)	-	2.54 (2.23, 2.89)	-	1.06 (0.55, 2.03)	-	1.15 (0.82, 1.60)
**Stroke**	N (%)	351 (1.23)	803 (2.81)	14 (0.50)	26 (0.93)	82 (1.04)	62 (0.79)
	Rate (CI)	-	6.12 (5.62, 6.65)	-	2.35 (1.52, 3.64)	-	1.84 (1.41, 2.39)
**MI or Stroke**	N (%)	620 (2.17)	1087 (3.81)	36 (1.28)	40 (1.43)	150 (1.91)	99 (1.26)
	Rate (CI)		8.50 (7.92, 9.13)		3.42 (2.38, 3.60)		2.93 (2.38, 3.60)

Previous: Event prior to the index date, Current: Event after the index date.

**Table 3:** **The hazard ratios (95% CIs) for cardiovascular events in patients treated with EBID or EBID + insulin compared to those treated with insulin, after adjusting for covariates, for all patients, and separately for patients without the history of cardiovascular diseases and without renal diseases before index date and during follow-up**

	**All patients**		**Without previous CVD**		**Without previous CVD & renal diseases**	
	**N = 39,225**		**N = 34,672**		**N = 33,744**	
	**HR (CI)**	**p-value**	**HR (CI)**	**p-value**	**HR (CI)**	**p-value**
**HF**						
**EBID vs Insulin**	0.34 (0.22, 0.52)	<0.001	0.34 (0.22, 0.52)	<0.001	0.32 (0.21, 0.50)	<0.001
**EBID + Insulin vs Insulin**	0.40 (0.32, 0.50)	<0.001	0.40 (0.32, 0.50)	<0.001	0.35 (0.28, 0.45)	<0.001
**MI**						
**EBID vs Insulin**	0.52 (0.23, 1.19)	0.12	0.24 (0.06, 0.99)	0.049	0.23 (0.06, 0.95)	0.043
**EBID + Insulin vs Insulin**	0.65 (0.44, 0.98)	0.038	0.52 (0.31, 0.91)	0.021	0.45 (0.28, 0.85)	0.011
**Stroke**						
**EBID vs Insulin**	0.50 (0.28, 0.84)	0.010	0.36 (0.18, 0.74)	0.005	0.37 (0.18, 0.75)	0.006
**EBID + Insulin vs Insulin**	0.38 (0.27, 0.54)	<0.001	0.27 (0.17, 0.42)	<0.001	0.26 (0.16, 0.42)	<0.001
**MI or Stroke**						
**EBID vs Insulin**	0.50 (0.32, 0.79)	0.003	0.33 (0.18, 0.63)	0.001	0.33 (0.18, 0.63)	0.001
**EBID + Insulin vs Insulin**	0.44 (0.34, 0.57)	<0.001	0.33 (0.23, 0.47)	<0.001	0.32 (0.22, 0.46)	<0.001

Adjusted for gender, ethnicity, age at the start of cohort, duration of diabetes, BMI, HbA1c, systolic and diastolic blood pressure on the index date, history of CV disease, any renal disease prior to index date or during follow-up, use of metformin (MET), sulphonylurea (SU), and CPMs or antihypertensive medications time.

Only 1% (n = 381) of patients had a history of MI, most in the insulin group (286 of 381). Among those without MI history (n = 38,844), rates of MI (per 1000 person years) in the insulin, EBID and EBID + insulin groups were 2.4, 0.95 and 0.99, respectively. Among patients without history of cardiovascular and renal diseases, compared to insulin group, patients in the EBID and EBID + insulin group had a 77% (HR CI: 0.06, 0.95) and a 55% (HR CI: 0.28, 0.85) reduced risk of MI, respectively, after adjusting for covariates (Table 3). Smoking and higher SBP was significantly associated with increased risk of MI (data not shown). Patients taking MET had a 44% reduced risk of MI (p < 0.01). However, the observed 27% increased MI risk associated with SU was not significant (p = 0.18). No significant risk reduction for MI was observed in these treatment groups for patients with a history of cardiovascular diseases.

Among 447 (1.1%) patients with a history of stroke, 351 (79%) belonged to the insulin group (Table 1). Overall, 891 (2.3%) patients had at least one event of non-fatal stroke during follow-up, with a rate (per 1000 person years) of 4.85; 90% (n = 803) of events occurred in the insulin group. The rate of stroke was nearly threefold higher in the insulin compared to the other treatment groups (Table 2). Among those without history of renal and cardiovascular diseases, compared to the insulin group, patients in the EBID and EBID + insulin groups had a 63% (HR CI: 0.18, 0.75) and a 74% (HR CI: 0.16, 0.42) reduced risk of stroke, respectively (Table 3). Higher SBP was significantly associated with increased risk of stroke (<0.001), whereas MET had a protective effect on stroke (HR CI: 0.61, 0.98). Treatment with EBID was associated with similar significantly reduced risk of composite event of MI or stroke (Table 3).

Among patients taking SU (n = 19282, 49%), the observed adjusted risk reductions for HF, MI, stroke and MI or stroke in the EBID and EBID + insulin groups were similar to those observed in the overall analysis. Among all patients, patients with renal diseases had 113% (p < 0.01) and 57% (p = 0.004) higher risk of HF and MI respectively. Renal disease was not associated with the risk of stroke (data not shown). HbA1c as a continuous measure or HbA1c above 58.5 mmol/mol (7.5%) at baseline was not significantly associated with the risk of cardiovascular events.

In our study cohort, 26052 (66.4%) patients were on metformin treatment. In patients treated with metformin, similar risk profile for MI, HF or stroke were observed in the EBID and EBID + insulin group, compared to those in the insulin group - the HR (95% CI; p) for composite of MI or stroke for EBID and EBID + insulin groups was 0.54 (0.34, 0.88; p = 0.012) and 0.44 (0.33, 0.59; p < 0.01) respectively.

The BMI at baseline in the insulin group was relatively lower than the other two groups (34 kg/m^2^) with 89% overweight or obese at baseline. We conducted additional analyses with baseline BMI categories with patients with “normal weight” as reference category. The risk for composite of MI or stroke in overweight and obese patients were not significantly higher compared to those in the normal weight category, after adjusting for other factors. Also, the rates/1000 person years of individual cardiovascular events were not significantly different by BMI categories (normal weight, overweight and obese) irrespective of treatment groups in the study cohort.

The mean (SD) and median (IQR) number of recorded insulin prescriptions during follow-up were 9 (9) and 7 (4, 13) respectively, with minimum median (IQR) 28 (6, 46) months of duration of insulin treatment in the insulin group. The mean (SD) and median (IQR) number of prescriptions in the exenatide group were 2.7 (1.7) and 2 (2, 3) respectively, with minimum median (IQR) 4 (3, 13) months of duration of exenatide treatment in the exenatide group. In the EBID + insulin group, the median (IQR) number of exenatide prescriptions was 3 (2, 4) and the minimum duration of exenatide treatment was 5 (3, 23) months. The minimum median (IQR) duration of insulin treatment in this group was 28 (2, 48) months. Longer duration of treatment with EBID was associated with significant reduced risk of HF, MI and stroke for analyses of patients with and without a history of cardiovascular diseases and renal diseases. Duration of insulin treatment was not associated with the risk of the cardiovascular events.

## Discussion

Our retrospective cohort study, based on a large age and HbA1c matched nationally-representative T2DM population data source with median follow-up of 3.5 years, showed that treatment with the GLP-1R agonist EBID with or without insulin was associated with reduced rates and risks of major macrovascular events compared with an insulin-treated cohort. This comparative longitudinal study demonstrated for the first time (to the best of our knowledge) that patients with T2DM who are treated with EBID, in addition to OADs, have lower rates and comparative risks of HF, MI and stroke than those treated with conventional combination treatment of insulin with OADs. As best as could be accounted for, cardiovascular risk reduction in patients treated with EBID was independent of variations in risk factors and comorbidities before and during follow-up.

Overall, 49% and 36% of patients had HbA1c above 58.5 mmol/mol (7.5%) and 63.9 mmol/mol (8%) respectively at the index date. We observed that patients with HbA1c above 58.5 mmol/mol (7.5%) in the EBID and EBID + insulin groups had significantly lower risks of HF and MI or stroke than analogous patients in the insulin group. Separate sub-group analyses for patients above and below an HbA1c of 63.9 mmol/mol (8%), patients without history of CV and renal diseases, and patients using MET/SU produced similar risk estimates for CV events in the EBID and EBID + insulin groups. These findings suggest that treatment with GLP-1R agonists may be associated with reduction in cardiovascular risk, when added with other conventional anti-diabetic drugs, after eliminating potential residual effects of other diseases, concomitant medications, and baseline glycaemic control.

Despite differences in follow-up time and comparator, observed event rates for HF, MI and stroke in our study were consistent with those reported by Best et al. [22,23]. Best et al. [22] evaluated the risk of MI, stroke and coronary revascularisation in patients treated with EBID versus other anti-diabetes drugs combined over a shorter period.

Mogensen et al. [24] reported significantly lower event rates for cardiovascular death and combined end point of MI, stroke and CV death among patients treated with DPP-4 inhibitors and GLP-1R agonist in addition to MET, compared to those treated with insulin and SU [24]. GLP-1R agonist in combination with MET was reported to have lower (but non-significant) risk ratio estimates for CV death, all cause mortality and combined end point during 2.1 years of median follow-up. In the absence of mortality data, our data are not directly comparable to that reported by Mogensen et al. However, in our subgroup analyses with patients taking MET and/or SU during median (IQR) 3.5 (2.0, 4.6) years of follow-up, the signal of risk reduction in patients treated with GLP-1R agonist is in line with the reported study.

Recent pre-clinical and basic science literature reports an array of beneficial findings with the GLP-1 receptor agonist exenatide [31-34]. These findings are broad and positively set the background for inflammatory state, cardiac function impacting both diastolic and systolic heart failure, peripheral vascular function and blood pressure control, and acute states of myocardial ischemia. Taken together, one could anticipate a positive impact on CV outcomes in a clinical setting, with the current epidemiological analysis suggestive of such a finding. Further clinical and epidemiological studies are needed to evaluate the possible extra-glycaemic benefits of treatment with GLP-1 receptor agonists.

The primary strength of this study was the large cohort of patients treated with EBID and/or insulin for the first time between 2005 and 2009 and followed for a median 3.5 years, with complete data on risk factors at the start of the cohort. Inclusion of an independent group of patients receiving both insulin and EBID or moving between these treatments was a novel aspect of this study and ensured that data on patients failing EBID were captured. Among 7870 patients in the EBID + insulin group, 70% initiated insulin treatment on or after the first prescription of exenatide. Patients initiating insulin before exenatide (n = 2334) had a significantly higher rate of HF per 1000 person years (rate: 9.01 vs 4.97). However, rates of MI and stroke were similar.

Another important strength of this study was extensive tracking of any CV or renal disease and reliable information on smoking status. Although it is not possible to account for all possible confounding factors in any longitudinal clinical epidemiological study, we adjusted for available relevant covariates in our risk analyses. Separate analyses were also conducted excluding patients with a history of CV disease and those with any renal disease. The large study population provided a representative source cohort that reflected common clinical practice for T2DM across the United States and provided adequate statistical power and information to adjust for potential confounding factors.

Patient-level data from electronic databases present challenges in terms of accuracy and completeness of the study variables of interest. Although we sought to mitigate potential sources of bias in the conduct of this study, some limitations remain, including potential misclassification of exposure and/or outcome, the potential for residual confounding, and aspects of competing risks. Exclusion of patients with incomplete information and thorough matching process may have resulted in selection of optimally-treated patients. Some residual confounding would be due to the non-availability of duration of diabetes data and data on socio-economic status, although we have adjusted for the pre-exposure time. Information on socio-economic status was likely to be relevant in this study given that treatment with EBID is more expensive and most likely that only individuals with good insurance would be able to afford it. Another important aspect is the failure to adequately adjust for the “bias due to indication”, which is very likely in the insulin-treated patients. Patients treated with insulin are generally deemed to have relatively poor health condition, which is difficult to adequately adjust for in clinical epidemiological studies, and we did not have complete data on the exact insulin doses to correctly adjust for it. Finally, the possibility of non-random prescription pattern leading to confounding by indication is impossible to overcome and cannot be excluded. However, we conducted analyses among patients without history of cardiovascular and renal diseases, and a balanced distribution of age and glycaemic level at the start of the cohort.

Diagnosis of CV disease using ICD-9 codes may not always represent a clinical diagnosis of the disease. However, the ICD-9 diagnosis-coded case definitions adhered to in this study have been applied in previous studies using the same or a similar database [35,36]. Prior studies have indicated that the estimated predictive value of administrative data for identifying CV endpoints is high (95% for acute MI and stroke). In studies based on administrative data, misclassification of events may be assumed to be non-differential with respect to exposure, providing a bias in the hazard estimate toward the null value that might obscure an association between exenatide and vascular risk. Another common weakness in pharmaco-epidemiological studies based on electronic primary care data, is the incompleteness on prescription information longitudinally for specific therapies. In our study, one of the primary inclusion criteria was to ensure a minimum of two consecutive prescriptions for insulin or exenatide, and the number of available prescriptions with dates during follow-up was well tracked. Due to the non-availability of mortality data, we could not conduct risk analyses on death due to cardiovascular diseases and all-cause mortality.

## Conclusions

Development of CV disease is one of the major complications of T2DM. The chronic hyperglycaemic state is often accompanied by dyslipidaemia, hypertension, low-grade systemic inflammation and oxidative stress, which collectively result in a high risk of micro- and macrovascular complications. Our population-based study provided evidence for the potential CV benefits of treatment with the GLP-1R agonist exenatide, while used in combination with conventional anti-diabetic therapies in reducing risks of HF, MI and stroke in patients with T2DM. While the confirmatory results from clinical trials are awaited, our epidemiological study provides much needed guiding information to the clinicians who are trying to balance the optimum glycaemic control along with management of CV risks in patients with T2DM.

## Abbreviations

ACE: Angiotensin-converting enzyme inhibitors
ARB: Angiotensin receptor blockers
BP: Blood pressure
CI: Confidence interval
CPM: Cardioprotective medication
CV: Cardiovascular
DPP-4: Dipeptidyl peptidase 4
EBID: Exenatide twice daily
GLP-1: Glucagon-like peptide-1
GLP-1R: Glucagon-like peptide-1 receptor
HbA1c: Glycated haemoglobin
HF: Heart failure
HR: Hazard ratio
ICD-9: International classification of diseases, ninth edition
IQR: Inter-quartile range
LVEF: Left ventricular ejection fraction
MET: Metformin
MI: Myocardial infarction
MQIC: Medical quality improvement consortium
OAD: Oral antidiabetes drugs
SD: Standard deviation
SU: Sulphonylurea
T2DM: Type 2 diabetes
US: United States
